# Radial endobronchial ultrasound-guided transbronchial biopsy for peripheral pulmonary malignancy: biopsy- or brushing-first?

**DOI:** 10.1186/s12890-019-0961-0

**Published:** 2019-10-31

**Authors:** Chun-Ta Huang, Yi-Ju Tsai, Chao-Chi Ho, Chong-Jen Yu

**Affiliations:** 1 Department of Internal Medicine, National Taiwan University Hospital, No. 7 Chung-Shan South Rd, Taipei, 100 Taiwan; 2 Graduate Institute of Clinical Medicine, College of Medicine, National Taiwan University, Taipei, Taiwan; 3 Graduate Institute of Biomedical and Pharmaceutical Science, College of Medicine, Fu Jen Catholic University, New Taipei City, Taiwan

**Keywords:** Biopsy, Brushing, Diagnosis, Endobronchial ultrasound, Peripheral pulmonary lesion

## Abstract

**Background:**

Radial endobronchial ultrasound (R-EBUS)-guided transbronchial biopsy (TBB) is a common diagnostic modality for peripheral pulmonary lesions; however, there is uncertainty about the optimal sequence of TBB and bronchial brushing during the procedure. Thus, we aimed to investigate whether a biopsy-first or brushing-first strategy confers a better diagnostic yield and safety signal for R-EBUS-guided procedures for peripheral pulmonary malignancy.

**Methods:**

From January 2017 to June 2018, consecutive patients referred for R-EBUS-guided TBB and bronchial brushing of peripheral pulmonary lesions and with a final malignant diagnosis were included. Patients were placed in a biopsy-first (biopsy followed by brushing) or a brushing-first (brushing followed by biopsy) group. The outcomes of interest were the diagnostic yield and complication profile of the procedures. Multivariate logistic regression and subgroup analysis were used to assess the impact of the procedure strategy.

**Results:**

A total of 438 patients were included and the diagnostic yield of R-EBUS-guided TBB plus brushing for peripheral pulmonary malignancy was 73%. The diagnostic yield was associated with the solid lesion appearance (odds ratio [OR] 2.01; 95% confidence interval [CI] 1.08–3.75) and R-EBUS probe position within the lesion (OR 1.92; 95% CI 1.08–3.42), and the yield rates were comparable between the biopsy-first and brushing-first strategies. Moreover, the safety signal did not differ between the two groups.

**Conclusions:**

The two procedure strategies were indistinguishable in terms of diagnostic efficacy and adverse events for patients with peripheral pulmonary malignancy. Current evidence indicates that in patients with peripheral pulmonary lesions suspected of being malignant, either biopsy-first or brushing-first is a viable and acceptable diagnostic strategy during R-EBUS-guided procedures.

## Background

Diagnosis of peripheral pulmonary malignancy can be attained via a variety of modalities, such as bronchoscopy, computed tomography (CT)-guided transthoracic needle biopsy and surgery. In the past two decades, advances in bronchoscopic procedures, such as radial endobronchial ultrasound (R-EBUS), virtual bronchoscopic navigation and electromagnetic navigation have made transbronchial biopsy (TBB) a more appealing and favorable approach [1, 2]. Of the above-mentioned techniques, R-EBUS-guided TBB provides a fair diagnostic yield and an excellent safety signal in diagnosing peripheral pulmonary malignancy [3–5]. In addition to TBB, auxiliary diagnostic tools, such as bronchial brushing and washing, are commonly used to reach the cytologic diagnosis, and combining these procedures may achieve a higher diagnostic yield than TBB alone [6, 7].

Besides the diagnostic modalities and tools, the details of the bronchoscopic procedures may affect their diagnostic yield. For instance, in bronchoscopically visible lesions, a brushing-first strategy provided a significantly higher diagnostic yield for lung cancer than a biopsy-first strategy [8]. In a similar clinical context, bronchial washing performed before or after endobronchial biopsy did not affect the diagnostic yield of biopsy and washing [9]. Regarding R-EBUS-guided TBB of peripheral pulmonary malignancy, both biopsy- and brushing-first strategies may be applied in clinical practice [10, 11]. However, to the best of our knowledge, no studies have been conducted to assess the ideal sequence of TBB and bronchial brushing during R-EBUS-guided procedures.

Therefore, the aims of this study were to investigate whether a biopsy-first or brushing-first strategy confers a better diagnostic yield and safety signal for R-EBUS-guided TBB of peripheral pulmonary malignancy.

## Methods

### Study setting and population

This study was conducted at National Taiwan University Hospital, a tertiary-care referral center in Taiwan. Consecutive adult patients who underwent R-EBUS-guided TBB of peripheral pulmonary lesions from January 2017 to June 2018 were screened for eligibility. A peripheral pulmonary lesion was defined as a lesion circumscribed by lung parenchyma and invisible through conventional bronchoscopy [12]. Criteria for inclusion in this study were (a) lesions with a final diagnosis of malignancy, either primary or metastatic and (b) both TBB and bronchial brushing performed during a single bronchoscopic session. The Research Ethics Committee of National Taiwan University Hospital approved the protocol and waived informed consent given the retrospective nature of the study and the lack of patient safety concerns.

### Bronchoscopic procedures

The bronchoscopic exam was primarily conducted by pulmonary fellows, as previously described, under the supervision of eight rotating pulmonary faculty in attendance [13, 14]. In brief, conventional bronchoscopy (BF-1 T260; Olympus Medical Systems Corp., Tokyo, Japan) was first performed to inspect the tracheobronchial tree after the patient received local anesthesia with lidocaine in the upper airway and intramuscular fentanyl for analgesia. Then, a 20-MHz radial-type ultrasonic probe (UM-S20-20R; Olympus Medical Systems Corp.), equipped with an endoscopic ultrasound center (EU-M30S; Olympus Medical Systems Corp.), was used to locate the peripheral pulmonary lesion, and R-EBUS-guided TBB and bronchial brushing were performed.

The biopsy was taken with a cup forceps (Micro-Tech Co. Ltd., Jiangsu, China), and was repeated until adequate tissue samples were collected. Bronchial brushing was performed with a 2-mm brush (ConMed Corp., New York, United States) and a few back-and-forth movements were used to retrieve the samples. The biopsy specimens were put in 10% formalin and transported to the histopathology laboratory for analysis. Tissue samples obtained by bronchial brushing were smeared onto glass slides, air-dried at room temperature and sent for cytology exam. During the study period, fluoroscopic guidance and rapid on-site evaluation (ROSE) were not utilized at our institution, and biopsy and brushing specimens were read independently by the pathologist and cytopathologist, respectively. A biopsy-first strategy (biopsy followed by brushing) or a brushing-first strategy (brushing followed by biopsy) was chosen by the in-charge faculty based on the day of the week on which the procedure was conducted. On Monday and Thursday, it would be a biopsy-first strategy, and on Tuesday and Wednesday, a brushing-first strategy.

### Data collection and follow-up

The primary outcome was the diagnostic yield of R-EBUS-guided TBB plus brushing for peripheral pulmonary malignancy; another outcome of interest was the incidence of procedure-related complications. Patient records and images were reviewed to obtain the following information: demographics, lesion size, location and appearance, absence or presence of a CT bronchus sign, probe position, procedure-related complications, and histopathologic and cytologic diagnosis. Lesion size was measured as the largest diameter on axial CT films. Lesion location was divided into five anatomic lobes. Lesion appearance was categorized as solid or non-solid (partly solid, pure ground-glass and cavitary). A CT bronchus sign was present if one or more bronchi leading directly to the peripheral pulmonary malignancy were identified on CT [15]. Probe position was classified as within, adjacent to or outside the peripheral pulmonary malignancy, as described previously [11]. Procedure-related complications included bleeding, pneumothorax, hemodynamic instability and bronchospasm. Self-limited bleeding was not counted as a complication in this study. Following R-EBUS-guided procedures, non-diagnostic lesions were subjected to CT-guided transthoracic needle biopsy, surgery, biopsy of other sites or repeat R-EBUS-guided TBB to pursue a definitive diagnosis of peripheral pulmonary malignancy.

### Statistical analysis

Between-group comparisons were performed using χ2 or Fisher’s exact test for categorical variables and independent samples t-test for numerical variables. A multivariate logistic regression model was constructed with the diagnostic yield of TBB plus brushing as the outcome variable predicted by the procedure strategy (biopsy-first vs. brushing-first), using all relevant covariates without model selection. To explore possible effect modification by lesion appearance, size and location, and probe position based on biologic plausibility, we used stratified analysis to estimate the diagnostic odds in each subgroup. *P* values < 0.05 were considered statistically significant and all tests were two-sided. All statistical analyses were performed using SPSS (version 20.0, IBM Corp.; Armonk, NY, US) or Stata (version 11, StataCorp.; TX, US).

## Results

### Study subjects

During the study period, a total of 438 patients with peripheral pulmonary malignancy were included for analysis. The average age of the study population was 66 ± 12 years, and slightly more than half of the subjects were male (*N* = 239, 55%). The mean diameter of the peripheral pulmonary malignancy was 37 ± 16 mm. An approximately equal number of malignant lesions were distributed between the upper lobes (*N* = 236, 54%) and the middle/lower lobes (*N* = 202, 46%), and the majority (*N* = 389, 89%) of them appeared solid on CT scans. Under most circumstances, the R-EBUS probe can be positioned within the malignant lesions (*N* = 375, 86%). The leading pathologic diagnoses in our study cohort were lung adenocarcinoma (*N* = 312, 71%), non-small cell lung cancer (*N* = 49, 11%) and lung squamous cell carcinoma (*N* = 36, 8.2%).

Table 1 shows the comparisons of clinical features between patients in the biopsy-first and brushing-first groups. The only characteristic that differed between the two groups was the probe location, i.e., the R-EBUS probe was more likely to be placed within the malignancy in the brushing-first group than in the biopsy-first group (90% vs. 81%, *P* = 0.004).

**Table 1:** Clinical characteristics and final diagnosis of the study population

Characteristics	Biopsy-first group	Brushing-first group	*P* value
	*N* = 219	*N* = 219	
Age, years	66 ± 13	65 ± 11	0.344
≥ 65	123 (56)	119 (54)	0.701
Gender			
Male	120 (55)	119 (54)	0.924
Lobar location			
Right upper lobe	63 (29)	52 (24)	0.547
Right middle lobe	18 (8.2)	13 (5.9)	
Right lower lobe	42 (19)	49 (22)	
Left upper lobe	56 (26)	65 (30)	
Left lower lobe	40 (18)	40 (18)	
Lesion size, mm	36 ± 16	37 ± 17	0.734
≤ 20	22 (10)	29 (13)	0.297
> 20	197 (90)	190 (87)	
CT appearance			
Solid	197 (90)	192 (88)	0.448
Non-solid	22 (10)	27 (12)	
CT bronchus sign			
Presence	215 (98)	216 (99)	0.999
Absence	4 (1.8)	3 (1.4)	
Probe location			
Within	177 (81)	198 (90)	0.004
Adjacent to or outside	42 (19)	21 (9.6)	
Lesion pathology			
Adenocarcinoma	162 (74)	150 (69)	0.656
Squamous cell carcinoma	18 (8.2)	18 (8.2)	
Small cell carcinoma	7 (3.2)	8 (3.7)	
Non-small cell carcinoma	22 (10)	27 (12)	
Metastasis	5 (2.3)	11 (5.0)	
Others	5 (2.3)	5 (2.3)	
Diagnostic yield	158 (72)	162 (74)	0.667

*CT* Computed tomography

### Diagnostic yield of TBB plus brushing

**Fig. 1:**
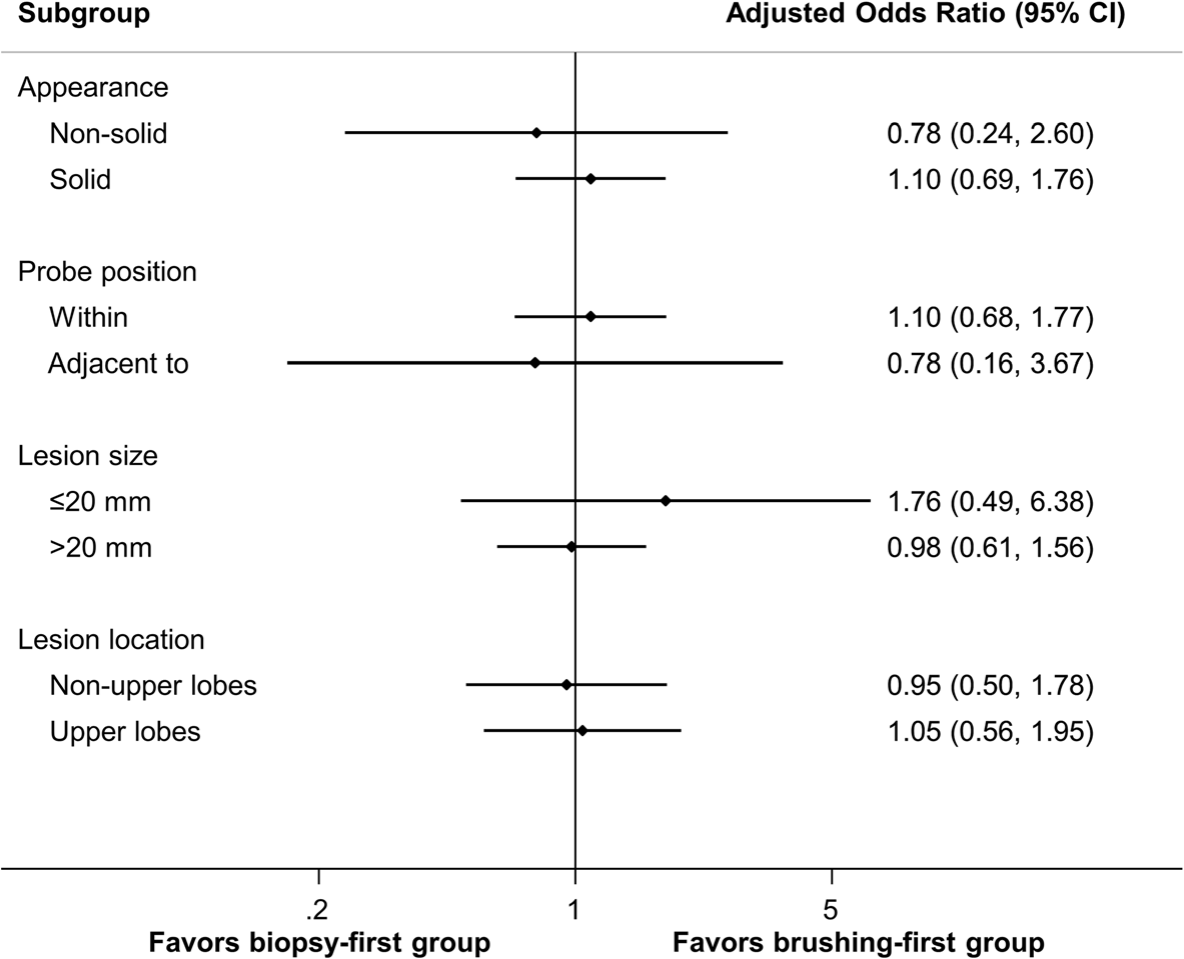
Subgroup analysis of the diagnostic yield of radial endobronchial ultrasound-guided transbronchial procedures in the biopsy-first group compared to the brushing-first group CI, confidence interval.

The overall diagnostic yield of TBB plus brushing for peripheral pulmonary malignancy was 73%. The diagnostic yield was associated with the CT appearance of the malignancy and probe location (Table 2). The diagnostic yield was not affected by the procedure strategy, lobar location, lesion size or pathology of the malignancy. In the multivariate analysis (Table 3), patients with solid peripheral pulmonary malignancy (odds ratio [OR] 2.01; 95% confidence interval [CI] 1.08–3.75) and an R-EBUS probe positioned within the lesion (OR 1.92; 95% CI 1.08–3.42) were more likely to have the diagnosis achieved by R-EBUS-guided procedures. Figure 1 shows the results of pre-specified subgroup analyses. The diagnostic yield of the R-EBUS-guided procedures did not vary significantly between the biopsy-first and brushing-first groups across all subgroups.

**Table 2:** Variables associated with the diagnostic yield of radial endobronchial ultrasound-guided transbronchial procedures

Characteristics	Diagnosed by R-EBUS-guided transbronchial procedures		*P* value
	No (*N* = 118)	Yes (*N* = 320)	
Procedure strategy			
Biopsy-first	61 (28)	158 (72)	0.667
Brushing-first	57 (26)	162 (72)	
Age, years			
< 65	50 (42)	146 (46)	0.544
≥ 65	68 (58)	174 (54)	
Gender			
Male	56 (48)	183 (57)	0.070
Female	62 (53)	137 (43)	
Lobar location			
Upper lobes	56 (48)	146 (46)	0.733
Non-upper lobes	62 (53)	174 (54)	
Lesion size, mm			
≤ 20	16 (14)	35 (11)	0.448
> 20	102 (86)	285 (89)	
CT appearance			
Solid	98 (83)	291 (91)	0.020
Non-solid	20 (17)	29 (9.1)	
Probe location			
Within	93 (79)	282 (88)	0.014
Adjacent to or outside	25 (21)	38 (12)	
Lesion pathology			
Adenocarcinoma	85 (72)	227 (71)	0.253
Non-small cell carcinoma	8 (6.8)	41 (13)	
Squamous cell carcinoma	11 (9.3)	5 (7.8)	
Small cell carcinoma	3 (2.5)	12 (3.8)	
Metastasis	7 (5.9)	9 (2.8)	
Others	4 (3.4)	6 (1.9)	

*CT* Computed tomography, *R-EBUS* Radial endobronchial ultrasound

**Table 3:** Multivariate logistic analysis of clinical features associated with the diagnostic yield of radial endobronchial ultrasound-guided transbronchial procedures

Variables		OR	95% CI	*P* value
Procedure strategy	Brushing-first vs. Biopsy-first	1.05	0.68–1.62	0.836
Appearance	Solid vs. Non-solid	2.01	1.08–3.75	0.029
Probe position	Within vs. Adjacent to or outside	1.92	1.08–3.42	0.026
Lesion size	> 20 mm vs. ≤20 mm	0.92	0.60–1.42	0.715
Lesion location	Non-upper lobes vs. Upper lobes	1.05	0.54–2.03	0.887

*CI* Confidence interval, *OR* Odds ratio

### Safety

No procedure-related mortality was observed in this study. Overall, complications occurred in 30 (6.8%) of the 438 patients (Table 4). The most commonly encountered complication was bleeding (*N* = 21, 4.8%), which was treated with instillation of topical epinephrine (*N* = 17) or bronchoscope wedge (*N* = 4). Other complications included pneumothorax (*N* = 6), unstable hemodynamics (*N* = 2) and bronchospasm (*N* = 1). Two R-EBUS-guided procedures were prematurely terminated due to the development of complications. The incidence of overall or individual complications was similar in both groups.

**Table 4:** Complications and their management during radial endobronchial ultrasound-guided transbronchial procedures

Events	Biopsy-first group	Brushing-first group	*P* value
	*N* = 219	*N* = 219	
Overall	16 (7.3)	14 (6.4)	0.705
Bleeding	12 (5.5)	9 (4.1)	0.502
Topical epinephrine	10 (4.6)	7 (3.2)	0.458
Wedging bronchoscope	2 (0.9)	2 (0.9)	0.999
Pneumothorax	3 (1.4)	3 (1.4)	0.999
Hemodynamic instability	1 (0.5)	1 (0.5)	0.999
Bronchospasm	0 (0)	1 (0.5)	0.999
Early terminated procedure	1 (0.5)	1 (0.5)	0.999

## Discussion

The present work is the first study to compare the diagnostic yield and complications of R-EBUS-guided TBB plus brushing for peripheral pulmonary malignancy using a biopsy-first or brushing-first strategy. The main findings of our study are as follows: (a) the overall diagnostic yield of TBB plus brushing of peripheral pulmonary malignancy was 73%; (b) a biopsy-first strategy provided diagnostic sensitivity for malignancy similar to a brushing-first strategy; (c) solid lesion appearance and position of the R-EBUS probe within the lesion were two features significantly associated with the diagnostic yield of TBB plus brushing; (d) the overall complication rate was 6.8%, with hemorrhage being the most common complication; (e) the safety signal did not differ between the biopsy-first and brushing-first groups.

The most important finding in this study is that the two strategies resulted in a comparable diagnostic yield for R-EBUS-guided procedures for peripheral pulmonary malignancy. In fact, most of the previous reports regarding TBB and bronchial brushing with the assistance of R-EBUS did not specify the sequence of the procedures [6, 16–18]. Kurimoto et al. adopted a brushing-first strategy for TBB and reported a high detection rate of 81% for peripheral pulmonary malignancy [11]; on the other hand, Roth et al. performed TBB prior to bronchial brushing and the diagnostic yield for malignancy was less than 50% [7]. A recent study by Hou et al. examined the optimal sequence of forceps biopsy and bronchial brushing of visible endobronchial lung cancer and found a significantly higher diagnostic rate (87%) in the brushing-first group compared to the biopsy-first group (79%) [8]. The authors rationalized their results by suggesting that endobronchial tumors can be more difficult to identify and sample after biopsy, since hemorrhage may contaminate the field available for brushing [8]. In contrast to previous experience with bronchoscopically visible lesions, our study showed a similar diagnostic yield for peripheral pulmonary malignancy in both the biopsy-first and brushing-first groups. One speculation is that with the guidance of R-EBUS, bronchial brushing of peripheral pulmonary lesions can be performed without visual aids, and post-biopsy bleeding will not obscure their visualization; thus, the procedure may be performed either before or after TBB. The other is that in experienced hands [4], a high quality R-EBUS-guided procedure could be performed to achieve a superior diagnostic sensitivity to peripheral pulmonary malignancy in both biopsy-first and brushing-first groups.

Our diagnostic sensitivity for peripheral pulmonary malignancy using TBB was consistent with that of previous studies of R-EBUS for malignant lesions, in which the diagnostic yield of R-EBUS ranged from 47 to 81% [6, 11, 16, 19–22]. The prevalence of malignancy present in the population being studied is a well-recognized explanation for the observed differences in the diagnostic sensitivity of R-EBUS-guided TBB for peripheral pulmonary lesions [5]. However, a wide variation in the yield of TBB still exists, even though only a subset of malignant lesions are chosen for evaluation in the literature. This heterogeneity may be explained by the discrepancies in other characteristics related to the cases, such as lesion size, personnel and institutional experience, and concomitant use of additional tools, like ROSE. The lowest diagnostic yield of 47% was observed in a study that included only solitary pulmonary nodules smaller than 20 mm [22], and undoubtedly, procedural experience improves the performance of R-EBUS-guided TBB [4]. Moreover, the application of ROSE has been shown to improve the diagnostic yield of TBB under R-EBUS guidance [23]. Therefore, it is important to consider patient and staff factors, and auxiliary modalities used when assessing how a certain technology like R-EBUS performs in clinical practice.

In a recent study by Chen, et al. [24], the diagnostic yield of R-EBUS-guided procedures for peripheral pulmonary lesions was positively correlated with the lesion size. Also, a meta-analysis found that lesion size was a significant determining feature in TBB performance [25]. However, the present study did not show such a finding. Our study population was composed solely of those with malignant pathology, and the procedures were conducted by a well-developed and experienced team. These specific settings may partly explain the discrepancy between our findings and others.

Safety is certainly a concern when choosing a modality or procedure. Consistent with prior studies [3, 26], a favorable safety profile with no mortality or sequelae with the use of R-EBUS-guided TBB for peripheral pulmonary malignancy was observed in this work. Pneumothorax is a well-known and potentially catastrophic complication after TBB, and our occurrence rate of 1.4% lies on the low end of those previously reported: 0 to 5.1% [3, 6, 10, 11, 17, 20–22, 26]. Bleeding is another major complication of TBB; however, its incidence is more difficult to compare across studies given the wide variation in definitions. On average, an incidence rate of 0.7% was reported for procedure-related bleeding regardless of its definition [11, 16, 17, 20–22, 26–28], but no major or serious bleeding events occurred with any patient in these reports. The bleeding rate observed in this study, 4.8%, seems to be higher than those of prior studies, but we adopted a stricter criterion for reporting this complication (any bleeding requiring further intervention) than have other studies (bleeding necessitating premature termination of the procedure or a bleeding amount of ≥30 ml) [11, 26]. With regard to our study aim of comparing safety signals between the biopsy-first and brushing-first strategies, the complication profiles and rates for R-EBUS-guided procedures were similar between the two groups of patients. Thus, the two procedure strategies studied herein were indistinguishable in terms of both efficacy and adverse events for patients with peripheral pulmonary malignancy.

In line with previous studies [4, 10, 11, 17, 18], ours found that probe position is a major determinant of the diagnostic yield of R-EBUS-guided TBB for peripheral pulmonary malignancy. This finding reinforces the importance of navigating the probe to the desired position that is visualized within the target lesion. In this study, the R-EBUS probe was more likely to be placed within the malignancy in patients in the brushing-first group, so the difference between probe positions may complicate the comparison of the diagnostic efficacy of the two study groups before adjusting for this confounder. However, in both the multivariate model and the subgroup analysis after taking probe position into consideration, the diagnostic sensitivity for peripheral pulmonary malignancy remained similar between the two strategies. This suggests the robustness of our study findings.

A couple of caveats pertaining to this study should be mentioned. First, the study findings represent a single-center experience with R-EBUS-guided TBB for peripheral pulmonary malignancy carried out by a well-developed bronchoscopy team; thus, the results may not be generalized to other settings, such as a less-experienced institution or a lower-level healthcare facility. Nonetheless, as the first study to deal with this issue, we hope our report will encourage more large-scale and elaborate studies in this field. Second, our study was retrospective, and as such, the choice of procedure strategy was at the discretion of the in-charge pulmonary faculty. In this regard, a selection bias may exist; however, baseline features of the participants were nearly balanced between the biopsy-first and brushing-first groups, and further statistical adjustment did not identify any significant differences in the study outcomes between the two groups.

## Conclusions

In summary, the timing of bronchial brushing, either before or after biopsy, for peripheral pulmonary malignancy, at least in experienced hands, did not influence the diagnostic yield of R-EBUS-guided TBB plus brushing. Moreover, a similarly favorable safety signal was observed between the two strategies. Therefore, current evidence indicates that in patients with peripheral pulmonary lesions suspected of being malignant, either biopsy-first or brushing-first would be a viable and acceptable strategy with respect to diagnostic sensitivity and safety during R-EBUS-guided procedures.

## Data Availability

The datasets used and/or analyzed during the current study are available from the corresponding author on reasonable request.

## Abbreviations

CI: Confidence interval
CT: Computed tomography
OR: Odds ratio
R-EBUS: Radial endobronchial ultrasound
ROSE: Rapid on-site evaluation
TBB: Transbronchial biopsy
